# The relationship between systemic iron homeostasis and erythropoiesis

**DOI:** 10.1042/BSR20170195

**Published:** 2017-11-29

**Authors:** Gautam Rishi, V. Nathan Subramaniam

**Affiliations:** The Liver Disease and Iron Disorders Research Group, Institute of Health and Biomedical Innovation, School of Biomedical Sciences, Queensland University of Technology, Brisbane, Queensland, Australia

**Keywords:** erythropoiesis, erythropoietin, hypoxia, hepcidin, iron metabolism, transferrin receptors

## Abstract

Red blood cell production (erythropoiesis) is the single largest consumer of iron in the body; this need is satisfied by maintaining a sensitive regulation of iron levels. The level of erythropoietic demand regulates the expression of the iron hormone hepcidin and thus iron absorption. Erythropoiesis-mediated regulation of hepcidin is an area of increasing importance and recent studies have identified a number of potential regulatory proteins. This review summarizes our current knowledge about these candidate erythroid regulators of hepcidin and the relation between transferrin receptors and erythropoiesis.

## Introduction

Red blood cells (RBCs) perform one of the most vital functions in the human body; the transport of oxygen to every tissue and organ. Oxygen binds to the iron atoms in the heme part of hemoglobin, the protein that makes up more than 90% of the dry content of RBCs [1]. The human body makes approximately 10 billion RBCs per hour and approximately the same number of RBCs die off [2], this makes RBC production (erythropoiesis) the largest user of iron in the body.

The other requirement for iron in erythropoiesis relates to its functional requirement in metabolically active and dividing cells. Iron acts as a cofactor for a number of enzymes including the DNA replicases, the three DNA polymerases (POLα, POLδ, and POLε) and DNA helicases (FANCJ) (reviewed in [3]) which are required for cell division in all actively dividing cells including the hematopoietic stem cells involved in erythropoiesis.

Kassebaum et al. [4] estimated that in 2010 approximately one third of the world’s population (32.9%) suffered from anemia. One of the predominant reasons for anemia is iron deficiency, and iron deficiency anemia (IDA) is described as one of the most common nutritional deficiencies worldwide [5]. The anemia associated with chronic disease (ACD) is another major form of anemia, which results from chronic conditions including chronic kidney disease, chronic inflammation, and cancer. On the other side of the spectrum are iron overload disorders, where excess iron is deposited in the parenchymal cells of the liver, heart, and pancreas and leads to a deterioration in their function. Thus, both excess and deficiency of iron are detrimental for health. The absence of a known mechanism to get rid of excess iron demands that iron homeostasis is tightly regulated.

RBCs develop from hematopoietic stem cells (HSCs); this process involves a number of steps and is regulated by the interplay between a number of cytokines, growth factors, and environmental cues [6]. The first erythroid progenitors that commit to the erythroid lineage are distinguished by their abilities to form erythroid colonies when grown on methyl cellulose; these progenitors are called the burst forming unit-erythroid (BFU-E) [7]. The BFU-E are the most immature cells which have committed to the erythroid lineage and differentiate into the more actively proliferating colony forming unit-erythroid (CFU-E) [7,8]. Apart from the differences in their proliferating capacity, the major distinction between these two progenitors is their requirement for different cytokines and growth factors. Whereas BFU-Es primarily require stem cell factor (SCF) [9] and some other growth factors e.g. insulin-like growth factor-1 (IGF-1), corticosteroids, interleukin-3 (IL-3), and interleukin-6 (IL-6), the CFU-Es are highly dependent on erythropoietin (EPO) [10]. The CFU-Es then differentiate into proerythroblasts that are morphologically distinct as the cells become committed to the erythroid lineage. The proerythroblasts do not contain much hemoglobin, but rapidly proliferate and hence require a constant supply of iron provided by transferrin [11,12]. The proerythroblasts then differentiate into the basophilic erythroblasts that start concentrating ribosomes in order to prepare for hemoglobin synthesis. The next stage is the polychromatic erythroblasts that stain deeply for hemoglobin and divide to give rise to the orthochromic erythroblasts that form the last nucleated stage of this cycle. The enucleation of the orthochromic erythroblasts results in the formation of reticulocytes. The reticulocytes exist in the blood for approximately 24 h remodeling their structure and altering their size before they finally adopt the distinct biconcave morphology of erythrocytes [13].

In the body, iron levels are regulated by modulating the levels of hepcidin, the iron regulatory hormone [14]. Hepcidin binds to the iron exporter protein ferroportin (FPN) and induces its degradation and internalization thus restricting the release of iron into the blood [15]. Iron is essential for a variety of different physiological processes and almost invariably all these processes regulate hepcidin directly or indirectly [16]. Some of these include the body iron levels, inflammation, hypoxia, and erythropoiesis. The focus of this review is to understand how erythropoiesis affects hepcidin regulation and the relationship between liver proteins involved in iron homeostasis and erythropoiesis.

## Erythropoietic regulation of hepcidin—finding the erythroid regulator

**Figure 1:**
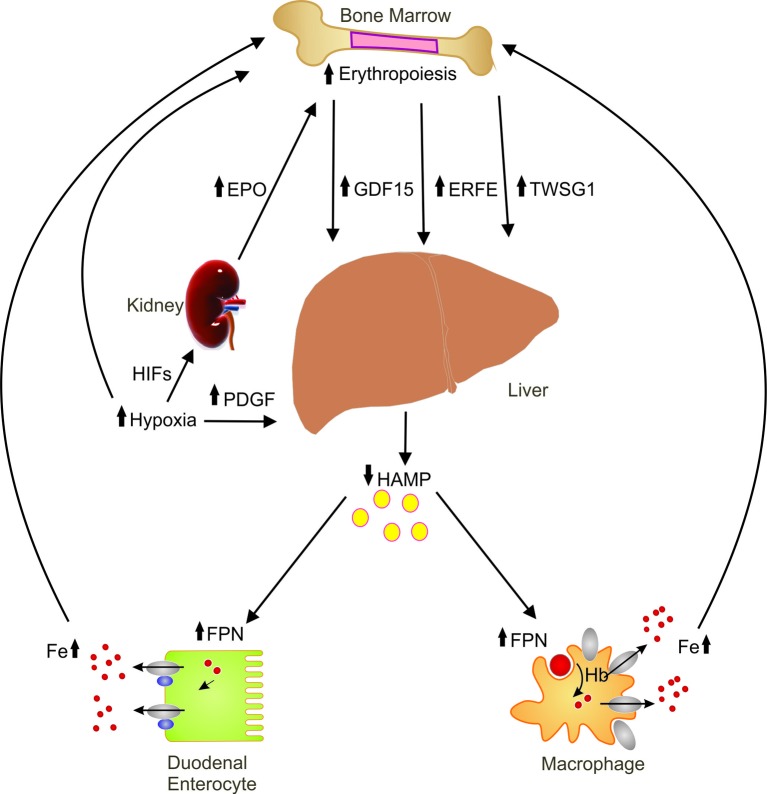
Erythroid regulation of hepcidin Schematic illustration of the candidate erythroid regulators and hypoxia on hepcidin regulation showing the various organs involved in this regulation. Abbreviations: EPO, erythropoietin; ERFE, erythroferrone; FPN, ferroportin; GDF15, growth and differentiation factor 15; HAMP, hepcidin; HIF, hypoxia inducible factors; PDGF, platelet-derived growth factor BB; TWSG1, twisted in gastrulation1.

Erythropoiesis is a known negative regulator of hepcidin [17,18]. Although the exact molecular details of this regulation are yet to be elucidated, several candidate regulators have been reported [19–21]. Since a proper erythropoietic response requires a constant supply of iron, it is believed that the erythroid compartment releases a soluble factor which limits the production of hepcidin in the liver [19]. Here we focus on the major players (Figure 1) thought to be involved in erythropoiesis-mediated regulation of hepcidin.

### Hypoxia inducible factors (HIFs)

Hypoxia links iron homeostasis to erythropoiesis. Oxygen transport utilizes the iron present in heme of the RBCs; in conditions of low oxygen availability, signals are sent to the bone marrow and hematopoietic compartment to increase RBC production. These signals are primarily mediated by HIFs, which regulate the transcription of a number of genes including EPO. HIF has an oxygen dependent α-subunit, either HIF1α [22,23], HIF2α [24] or HIF3α [25], and a constitutively expressed β-subunit, aryl hydrocarbon nuclear translocator (ARNT) [26].

In normoxic conditions the oxygen-dependent HIFα subunits are degraded by prolyl hydroxylase domain-containing enzymes (PHD). These enzymes need both iron and oxygen in order to be activated, forming an important link between iron and oxygen concentrations. The active PHDs are responsible for hydroxylating the HIFα’s at their proline residues, which makes them susceptible for ubiquitination. In hypoxic conditions, PHDs become inactive thus increasing the stability of HIFα. These subunits then bind to ARNT and other transcription factors to regulate the transcription of genes in response to hypoxia.

Mice with a hepatocyte-specific deletion of HIF1α do not respond to iron deficiency or hypoxia by decreasing hepcidin expression as is seen in wild-type (WT) mice [27]. HIF1α also binds to three hypoxia responsive elements in the hepcidin promoter and reduces its expression [27]. HIF2α on the other hand seems to be involved in regulating iron uptake, at least in the intestinal epithelium [28].

Hypoxia may also be acting indirectly in iron homeostasis by interrupting the expression of molecules involved in hepcidin regulation. Hypoxia and iron deficiency were shown to increase the mRNA levels of transmembrane serine protease 6 (TMPRSS6) [29]. TMPRSS6, also known as matriptase 2, is a known negative regulator of hepcidin. TMPRSS6 cleaves membrane hemojuvelin (HJV) at an external site [30]. The cleavage of HJV results in a disruption of bone morphogenetic protein-sma and mothers against decapentaplegic (BMP-SMAD) signaling, leading to a decrease in hepcidin transcription. Patients with mutations in *TMPRSS6* [31] develop a rare form of anemia known as iron-refractory iron-deficiency anemia (IRIDA) which is characterized by high levels of hepcidin due to a constitutive activation of the BMP–SMAD pathway, as the TMPRSS6 protein is unable to cleave HJV. Another molecule that hypoxia may be indirectly affecting hepcidin is through furin. Hypoxia also increases *furin* mRNA levels through HIF1α in HepG2 and Hepa1-6 (mouse hepatoma) cells [32]. Furin cleaves HJV to form soluble HJV [33], sHJV acts as an antagonist to inhibit the BMP–SMAD pathway resulting in a reduced hepcidin expression.

Hypoxia also induces the expression of EPO in the kidney and EPO was also thought to be one of the candidates for erythropoiesis-mediated regulation of hepcidin. It appears that this hypoxia-mediated repression of hepcidin requires EPO; mice where EPO was deleted were unable to reduce hepcidin expression in response to hypoxia [34]. A separate pathway of hypoxia-mediated suppression of hepcidin has been proposed to act through the platelet-derived growth factor (PDGF)-BB [35]. PDGF-BB levels in the serum of hypoxic individuals were correlated to hepcidin levels, and treatment of mice with a PDGF receptor inhibitor abrogated the hypoxia-mediated down-regulation of hepcidin in mice [35].

### EPO

An increase in erythropoietic demand also leads to an increase in the production of EPO, and it was shown that an increase in erythropoietic activity was a negative regulator of hepcidin [17,18,36]. These studies and others suggested that EPO was the mediator of hepcidin reduction in response to erythropoiesis. Subsequent studies in mice were able to show that in the absence of erythropoietic activity (pretreatment with carboplatin or doxorubicin) EPO could not suppress hepcidin [37]. It became apparent that EPO may not be directly involved in hepcidin regulation and this contributed to the development of the theory that there is a secreted, soluble, factor which mediates this increased iron demand from the erythroid compartment to the liver.

### Growth differentiation factor 15 (GDF15) and twisted in gastrulation 1 (TWSG1)

Two ‘erythroid regulator’ candidate genes were identified by performing a microarray analysis on RNA isolated from primary erythroblasts donated by normal subjects [19]. This resulted in the identification of two secreted proteins that are members of the TGF family of proteins—GDF15 and TWSG1 [19]. Sera from β-thalassemic patients were able to down-regulate *HAMP* in primary hepatocytes, but this reduction was not observed in the absence of GDF15 [19]. This was compelling evidence for GDF15 to be the erythroid factor; other studies also found an increase in GDF15 levels in patients with pyruvate kinase deficiency [38], multiple myeloma [39], and anemia [40]. This increase in GDF15 levels was not as high as observed in β-thalassemic patients and in some cases, there was a positive correlation between GDF15 expression and hepcidin levels [40]. *Gdf15* knockout mice which were subjected to two consecutive phlebotomies and did not exhibit any differences in hepcidin levels as compared with WT mice; these results suggested that either GDF15 was not the putative erythroid factor or its effects could be overcome by other pathways involved in hepcidin regulation [41].

Unlike GDF15, TWSG1 has not received as much attention and the only study which suggested that it could be a factor secreted by early erythroblasts capable of regulating hepcidin was published in 2009 [20]. TWSG1 acts through the BMP–SMAD pathway, levels of pSMAD1/5 were decreased in cells treated with TWSG1. Interestingly, this down-regulation was synergistic with a treatment with BMP2 and BMP4 [20]. *Gdf1*5 mRNA levels increased in the bone marrow and spleens of five different models of anemia whereas *Twsg1* showed no change as compared with controls [42]. These studies indicate that GDF15 may have a role in the erythropoiesis-mediated down-regulation of hepcidin but it is highly unlikely that TWSG1 does.

### Erythroferrone (ERFE)

Recently, there has been another addition to this list of potential erythroid regulators of hepcidin: the product of the *Fam132b* gene, known as erythroferrone (*ERFE*) [21]. EPO injections resulted in an increase in mRNA expression of *ERFE* in the erythropoietic organs (bone marrow and spleen) [43]. *Fam132b^−/−^* mice do not suppress hepcidin in response to phlebotomy as strongly as WT mice [43]. Phenylhydrazine (PHZ) injections induced blood loss and a strong induction of EPO, this resulted in an increase in the mRNA expression of *Fam132b* [44]. As expected the increase in erythropoiesis was followed by a decrease in hepcidin, but this suppression was reduced in mice where ERFE was knocked down using lentiviral vectors, suggesting that EPO and erythropoiesis-mediated regulation of hepcidin requires ERFE [44]. ERFE also seems to play a role in anemia of inflammation. *Fam132b^−/−^*mice injected with *Plasmodium berghei* K173 were not able to suppress hepcidin in response to the anemia induced by malaria [45]. Similarly, *Fam132b^−/−^* mice injected with *Brucella* were able to recover faster from anemia as they did not suppress hepcidin as efficiently as WT mice [46]. These results indicate a wider role for ERFE in hepcidin regulation and add more evidence to its role as the erythroid regulator.

## Erythropoiesis and transferrin receptors

### Transferrin receptor 1 (TFR1)

Transferrin receptor 1 is a ubiquitously expressed protein that is responsible for the uptake of transferrin (TF)-bound iron. This mechanism of iron uptake is the most common among all cells of the body; it is also essential during development as the loss of *Tfr1^−^*in mice leads to embryonic lethality [47]. The homozygote KO embryos showed signs of severe anemia and hypoxia underlining the importance of TF-bound iron uptake mechanisms. The process of erythropoiesis itself involves very rapid proliferation, and at each stage there are considerable transcriptomic changes taking place [48]. These cells would thus be metabolically very active, with increasing the demands for iron, hence iron becomes an integral part of the developmental process itself.

The binding of EPO to the erythropoietin receptor (EPOR) results in the activation of Janus activated kinase 2 (JAK2) which in turn phosphorylates the cytoplasmic tail of EPOR [49]. The activated phosphorylated tail of EPOR becomes a signal transducer for many other pathways such as signal transducer and activator of transcription 5 (STAT5) [49]. Once activated the phosphorylated STAT5 is then translocated to the nucleus where it leads to activation or repression of its target genes. One of the downstream target genes affected by Stat5 activation is *Tfr1*; mice lacking Stat5 had less Tfr1 on the cell surface of erythroid cells. *Tfr1* also has three Stat5 binding sites in its first intron [50].

In addition to erythropoiesis-mediated regulation of *Tfr1*, body iron levels can also regulate *Tfr1* mRNA levels through the iron responsive element (IRE)–IRE binding proteins (IRP). The IRE–IRP system is mediated by the binding of the RNA-binding proteins (IRPs) to a conserved sequence in the 5′- or the 3′-untranslated region (UTR) of the mRNA, thus either inhibiting translation or stabilizing the mRNA [51]. This conserved sequence in the RNA is known as the iron responsive element (IRE) and forms a stem–loop structure to which the IRPs bind [51].

*Tfr1* regulation in erythroid cells may also be heme-dependent and iron-independent, suggesting an erythroid-specific regulation of iron uptake [52]. When differentiating mouse erythroleukemia (MEL) cells were treated with iron chelators, they did not show a change in Tfr1 expression. Similarly there was no change in the levels of Tfr1 when the levels of iron were increased in the differentiating MEL cells [52]. On the other hand, treatment with heme synthesis inhibitors, decreased TFR1 [52,53], indicating a heme-specific and IRP-independent regulation of TFR1. Recently it was shown that polymeric immunoglobulin A can bind to TFR1 and act synergistically with EPO to increase erythropoiesis in mice [54]. These studies have established another layer of interplay between erythropoiesis and systemic iron homeostasis where erythropoiesis is able to manipulate levels of a receptor involved in iron uptake to meet the increased iron demands.

### Transferrin receptor 2 (TFR2)

The homolog of TFR1, TFR2 is thought to be involved more in the regulation of iron than its uptake. Patients [55] and mice [56] with defects in TFR2 develop iron overload. *TFR2* was mapped to chromosomal position 7q22 in the human genome and 5qG2 in the mouse genome, and is close to *EPO* (on the opposite strand). An upstream analysis of the promoter region of TFR2 revealed binding sites for several erythroid-specific transcription factors [57]. There are two GATA1 binding sequences in both human and mouse DNA, -52 to -57 and -23 to -19 in mouse, and -61 to -56 and -26 to -22 in the human DNA [57]. Two putative C/EBP binding sequences were also found in both humans and mice, the first around -240 and the other in a reverse orientation at around -190 [57]. In addition to this, several CACCC consensus sequences were detected within 1 kb upstream of the *TfR2* promoter, these are potential erythroid Kruppel-like factor (EKLF) binding sites (another erythroid-specific transcription factor) [57]. It was shown that both EKLF and GATA1 increase the luciferase activity of the *TFR2* promoter, although in one case where a shorter promoter was used EKLF decreased the activity suggesting it has an inhibitory effect [57]. These observations suggest that *TfR2* could be regulated in an erythroid-specific fashion.

Genome wide association studies have identified associations between the *TFR2–EPO* locus and various hematological traits including RBC number and hematocrit [58,59]. *TFR2* expression is lower in patients with high risk myelodysplastic syndromes (MDS), such as refractory anemia with excess blasts type 2 (RAEB2) [60]. Increased levels of *TFR2* are associated with higher survival rates in patients with acute myeloid anemia [61].

The erythroid function of TFR2 was further supported by the demonstration that it may be required for the proper localization of EPOR [62]. Erythroid progenitor cells from young *Tfr2*-deficient mice had a reduced colony forming ability even in the presence of physiological concentrations of EPO [62]. Further evidence for this erythroid-specific function has been highlighted by recent mouse models generated by us [63,64] and others [65–67]. Using these models, it has been suggested that Tfr2 is required for maintaining a proper erythropoietic response in stress conditions (dietary iron deficiency or genetic anemia). This has opened a novel link between iron and erythropoiesis, where a molecule may be performing a dual role in specific conditions. The mechanism of how Tfr2 contributes to erythropoiesis is yet to be identified although there have been suggestions of a soluble form of Tfr2 which is released only in iron-deficient conditions [68], or increased EPO sensitivity of the erythroid cells in the absence of Tfr2. Further studies will be required to completely understand this dual function of TFR2.

In summary, the mechanisms and molecules discussed above point to a very tightly regulated and sensitive erythroid control over iron homeostasis. This is mediated through regulating hepcidin and molecules involved in systemic iron homeostasis e.g. TFR1. Additional studies on the role of ERFE are no doubt underway and will contribute to defining its role as the erythroid regulator. Further studies should concentrate on the role of ERFE in human erythropoietic disorders and its role in hepcidin regulation.

## Abbreviations

ACD: anemia associated with chronic disease
ARNT: aryl hydrocarbon nuclear translocator
BFU-E: burst forming unit-erythroid
BMP-SMAD: bone morphogenetic protein-sma and mothers against decapentaplegic
CFU-E: colony forming unit-erythroid
EKLF: erythroid Kruppel-like factor
EPO: erythropoietin
EPOR: erythropoietin receptor
ERFE: erythroferrone
GDF15: growth differentiation factor 15
HIF: hypoxia inducible factor
HJV: hemojuvelin
HSC: hematopoietic stem cell
IDA: iron deficiency anemia
IGF-1: insulin like growth factor-1
IL-3: interleukin-3
IL-6: interleukin-6
IRE: iron responsive element
IRIDA: iron-refractory iron-deficiency anemia
IRP: IRE binding proteins
JAK2: Janus activated kinase 2
MDS: myelodysplastic syndromes
MEL: mouse erythroleukemia
PHD: prolyl hydroxylase domain-containing enzymes
PHZ: phenylhydrazine
POL: polymerases
RAEB2: refractory anemia with excess blasts type 2
RBC: red blood cell
SCF: stem cell factor
STAT5: signal transducer and activator of transcription 5
TF: transferrin
TFR: transferrin receptor
TMPRSS6: transmembrane serine protease 6
TWSG1: twisted in gastrulation 1
WT: wild-type
